# Effect of AAA Size on Mortality and Morbidity After Endovascular Aortic Repair

**DOI:** 10.3390/jcm14165787

**Published:** 2025-08-15

**Authors:** Paulina Julia Wiatrzyk, Oliwia Grzelak, Joanna Halman, Klaudia Szydłowska, Jacek Wojciechowski

**Affiliations:** 1 Students Scientific Circle of Vascular Surgery, Medical University of Gdańsk, 80-210 Gdańsk, Poland; 2 Department of Vascular Surgery, University Clinical Centre in Gdańsk, 80-952 Gdańsk, Poland

**Keywords:** abdominal aortic aneurysm, aneurysm diameter, endovascular aneurysm repair, stent-graft, complications, endoleak, outcome analysis, individualized risk stratification, precision medicine in vascular surgery, imaging-derived biomarkers

## Abstract

**Objectives:** To analyze the effect of abdominal aortic aneurysm (AAA) diameter on late complication occurrence and survival in patients following endovascular aneurysm repair (EVAR). **Methods:** The study was a retrospective cohort analysis with a prospective follow-up of 176 patients diagnosed with unruptured AAA who underwent EVAR from 2016 to 2024. Preoperative computed tomography (CT) images were used to measure maximal aneurysm diameter. Prospective follow-up data were collected post-EVAR at 1 month, 6 months, and annually through clinical evaluations and imaging studies. The mean follow-up duration was 26 months. For statistical purposes, the group was divided into tertiles based on aneurysm size. This study was intentionally focused on aneurysm size as an isolated imaging parameter, and did not incorporate other known predictors of complications, such as neck morphology or device-related variables. As such, key limitations include the single-center design, relatively small sample size, and lack of data on anatomical features beyond maximum diameter. **Results:** Kaplan–Meier survival analysis demonstrated that patients in the highest tertile of aneurysm size experienced significantly higher rates of long-term adverse outcomes compared to those in the lower two tertiles, both in terms of late complications (log-rank *p* = 0.049) and all-cause mortality at 36 months (*p* = 0.022). In multivariate logistic regression, the occurrence of late complications was independently associated with symptomatic presentation (*p* = 0.003, OR = 3.616, 95% CI: 1.533–8.529) and acute admission (*p* = 0.033, OR = 0.345, 95% CI: 0.130–0.916). The largest aneurysms were significantly associated with late endoleak (*p* = 0.041, OR = 5.365, 95% CI: 1.071–26.871). **Conclusions:** This study demonstrates that AAA size is an independent predictor of both late complications and long-term survival following EVAR. Patients with larger aneurysm diameters experienced significantly higher rates of late complications and reduced overall survival.

## 1. Introduction

Endovascular aneurysm repair (EVAR) has become the predominant treatment strategy for abdominal aortic aneurysm (AAA), particularly in elderly patients with multiple comorbidities and favorable anatomy. In many vascular centers, EVAR now accounts for the vast majority of AAA repairs, often approaching 90%, and open surgical repair is increasingly rare [1]. This shift, while reflecting the minimally invasive appeal and perioperative safety profile of EVAR, raises concerns about long-term durability, surgeon experience and training in open techniques, and appropriate patient selection in an era where open repair is becoming a lost art among European centers [2,3,4,5].

The relationship between aneurysm size and patient outcomes remains complex and requires a thorough understanding. The existing literature presents conflicting evidence regarding the prognostic value of aneurysm size in patients undergoing EVAR. While some studies have identified a larger preoperative aneurysm diameter as a risk factor for increased mortality following EVAR, others have demonstrated that although aneurysm size may influence midterm survival, it does not consistently emerge as an independent predictor of long-term mortality [6,7,8]. This inconsistency may partly result from methodological heterogeneity and unaccounted confounding factors. Differences in patient comorbidities, anatomical complexity, device selection, perioperative management, and duration of follow-up could all influence outcomes independently of aneurysm size. Furthermore, variations in statistical modeling and the adjustment for these confounders may partially explain why some studies identify aneurysm diameter as an independent predictor, while others do not. This ambiguity raises essential clinical questions, particularly in light of the persistently high rate of late complications, such as endoleaks, reinterventions, and aneurysm sac expansion, observed in patients treated with EVAR [9]. It also challenges current practice paradigms, where even very large aneurysms are often managed with EVAR despite uncertain long-term benefit [10]. As vascular surgery transitions toward individualized, evidence-based decision-making, a more nuanced understanding of how imaging-derived markers, such as aneurysm size, influence long-term morbidity and mortality is crucial [11]. Such insights could help tailor treatment strategies, identifying patients who may benefit from more intensive surveillance, adjunctive techniques, or, in select cases, consideration of open surgical repair rather than routine stent-graft placement. Ultimately, integrating aneurysm size into a comprehensive, personalized risk stratification model may improve the safety, durability, and outcomes of aneurysm treatment in contemporary practice [12]. This study seeks to clarify whether maximum aneurysm diameter serves as an independent predictor of long-term complications and mortality following EVAR, beyond its established role in rupture risk.

## 2. Materials and Methods

### 2.1. Study Design

This study was a retrospective cohort analysis with prospective follow-up conducted on patients diagnosed with AAA who underwent EVAR at the Department of Vascular Surgery of the Medical University of Gdańsk, Poland, from 2016 to 2024. The study was approved by the Local Ethics Committee (approval no KB/247/2025, issued on 8 May 2025). The study design followed established principles of retrospective cohort analysis and post-EVAR surveillance protocols as described in previous literature [13,14].

### 2.2. Patient Population

**Figure 1:**
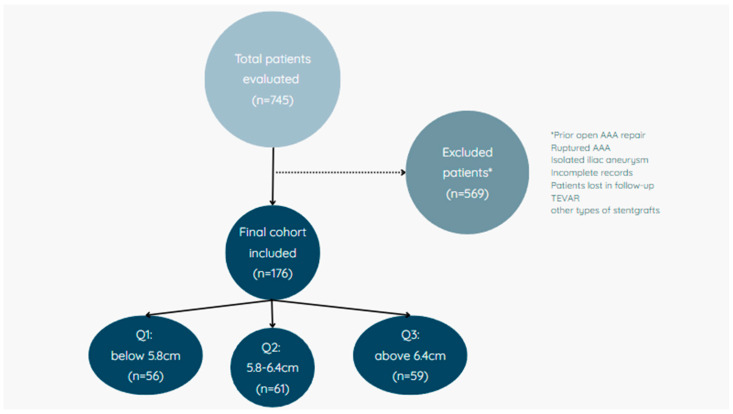
Study population flow diagram.

A total of 176 patients with AAA who underwent EVAR were included in this study. Patients were selected based on the following inclusion criteria: (1) a confirmed diagnosis of AAA via computed tomography angiography (CTA) scan, (2) having undergone EVAR treatment within the IFU, and (3) the availability of complete medical records. Exclusion criteria comprised prior open surgical repair of the aneurysm, disqualification from endovascular intervention due to significant comorbidities contraindicating treatment, and incomplete follow-up data. Patients who underwent EVAR for ruptured AAA or isolated iliac artery aneurysm were also excluded. For statistical purposes, the group was divided into tertiles based on aneurysm size. Based on this classification, size thresholds were established: Q1, below 5.8 cm; Q2, 5.8–6.4 cm; and the group of patients with the largest aneurysms: Q3, above 6.4 cm. Q3 was compared to the combined group of Q1 and Q2 (Figure 1). Tertile thresholds were defined based on the cohort’s internal size distribution and were not aligned with fixed clinical thresholds. This approach allowed stratified outcome analysis while acknowledging inter-individual variability in aneurysm anatomy.

### 2.3. Data Collection

**Figure 2:**
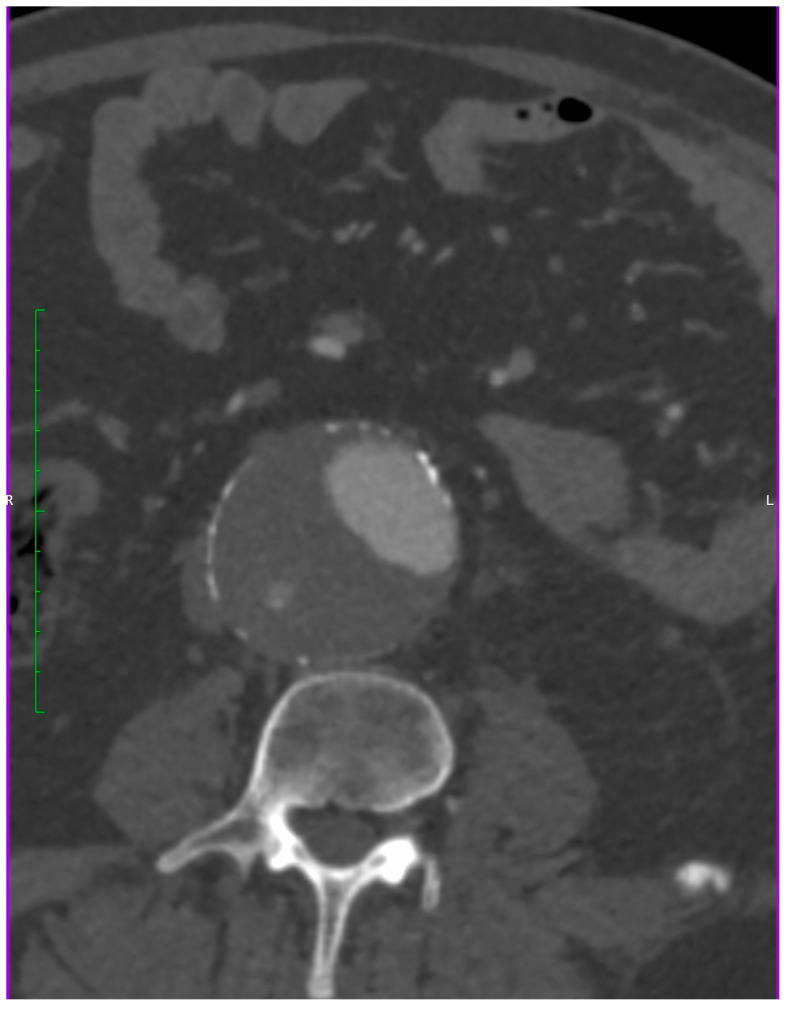
Measurement of an aneurysm using OsiriX software.

Retrospective data were extracted from patient electronic medical records, including demographic information (age, sex, body mass index), comorbidities (coronary artery disease (CAD), arterial hypertension, diabetes mellitus (DM), history of myocardial infarction, coronary revascularization, hyperlipidemia, heart failure (HF), chronic obstructive pulmonary disease (COPD), peripheral artery disease (PAD), chronic kidney disease (CKD), dialysis, transient ischemic attack (TIA), stroke, hypothyroidism, history of smoking cigarettes) aneurysm characteristics, as size, morphology, location, using OsiriX (Pixmeo SARL, Genev, Switzerland ver 11.0) software—a validated tool used in prior vascular imaging studies and indications for surgical intervention. Preprocedural CT scans were reviewed to measure the maximum diameter of the aneurysm in the horizontal plane and assess for any anatomical considerations that might affect EVAR (Figure 2). All measurements were performed by two independent observers (PW and OG), both trained in vascular imaging analysis. In cases of discrepancy greater than 2 mm, a joint review was conducted to reach consensus. This process was included to reduce inter-observer variability. Procedure data included differentiation to EVAR or percutaneous EVAR (pEVAR) with or without additional iliac branch, percutaneous transluminal angioplasty (PTA), endarterectomy, or stent application in a different artery than the abdominal aorta during the same procedure. Prospective follow-up was standardized and conducted at 1 month, 6 months, and annually thereafter. Data were gathered through clinic visits and imaging studies (CT or duplex ultrasound), in order to monitor for complications, including endoleaks, stroke, myocardial infarction, reinterventions, increase in aneurysm size, major bleeding, aneurysm rupture, and death.

### 2.4. Statistical Analysis

Statistical analyses were performed using IBM SPSS Statistics 30. Descriptive statistics were provided for all variables. Continuous variables were presented as mean ± standard deviation (SD) or median with interquartile range (IQR). Categorical variables were expressed as frequencies and percentages. The relationship between AAA size and outcomes was analyzed using regression models, adjusting for potential confounders, including age, sex, and comorbidities. Kaplan–Meier survival analysis was utilized to assess long-term survival, with log-rank tests applied to compare survival curves among different aneurysm size categories. A significance level of *p* < 0.05 was set for all statistical tests.

## 3. Results

### 3.1. Patient Demographics and Comorbidities

**Figure 3:**
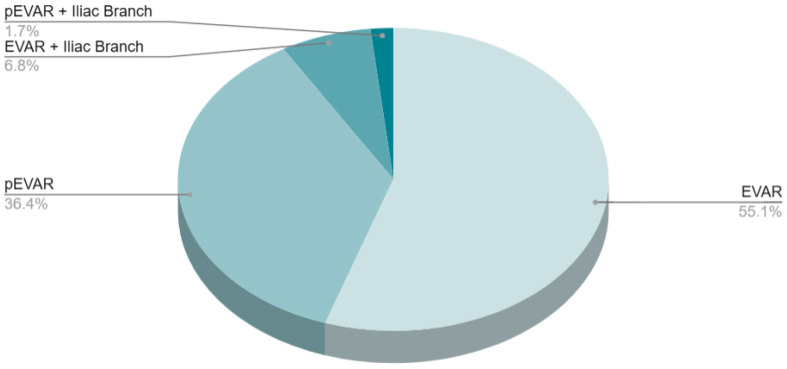
Distribution of procedure types among EVAR interventions; pie chart.

The cohort consisted of 32 women and 144 men; the mean age of the participants was 74.2 years, with a standard deviation of 7.4 years. The smallest preoperative diameter was 45 mm, and the largest AAA was 105 mm. Comorbidities are presented in a table and a pie chart below (Table 1; Figure 3).

### 3.2. Types and Distribution of EVAR Procedures

All patients who underwent EVAR received one of four types of procedures: EVAR (55.11%), pEVAR (36.36%), EVAR with iliac branch (6.82%), or pEVAR with iliac branch (1.7%) (Table 2).

### 3.3. Survival and Adverse Events Stratified by Aneurysm Size

**Figure 4:**
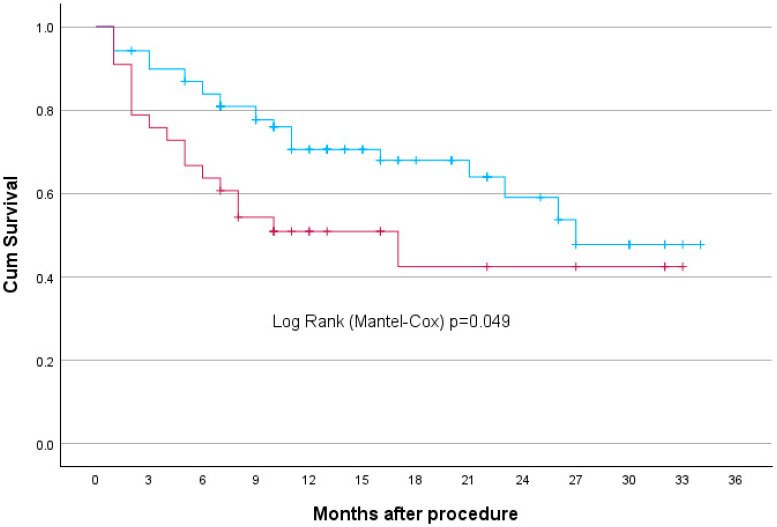
Kaplan–Meier curve—AAA size correlation with late complications (endpoint 36 months). The blue line represents Q1 and Q2, while the red line represents Q3.

**Figure 5:**
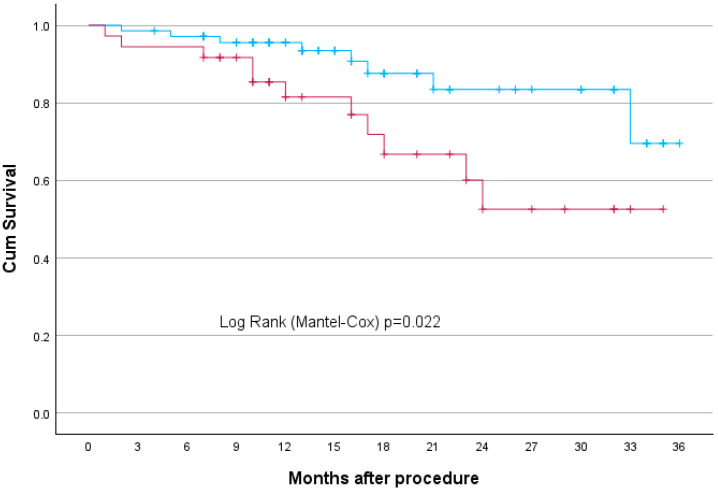
Kaplan–Meier curve—AAA size correlation with death (endpoint 36 months). The blue line represents Q1 and Q2, while the red line represents Q3.

Kaplan–Meier survival analysis revealed that Q3 patients had significantly higher rates of long-term adverse outcomes compared to Q1 and Q2 combined, both for late complications (*p* = 0.049) (Figure 4) and overall mortality (*p* = 0.022) (Figure 5) at 36 months. Long-term adverse events included in the study were: MI, stroke, endoleak, wound infection, aneurysm rupture, need for reintervention, and death.

The largest aneurysms were significantly associated with all types of late endoleak (*p* = 0.041, OR = 5.365, 95% CI: 1.071–26.871).

Logistic regression identified key predictors of complications. Early complications were significantly influenced by the type of stent-graft used during EVAR—iliac branch devices were linked to an increased risk of early complication occurrence (*p* = 0.008, OR = 5.884, 95% CI: 1.598–21.662). Late complications were significantly correlated with symptomatic presentation (*p* = 0.003, OR = 3.616, 95% CI: 1.533–8.529) and acute admission (*p* = 0.033, OR = 0.345, 95% CI: 0.130–0.916).

### 3.4. Correlation Between Aneurysm Diameter and Hospital Stay

**Figure 6:**
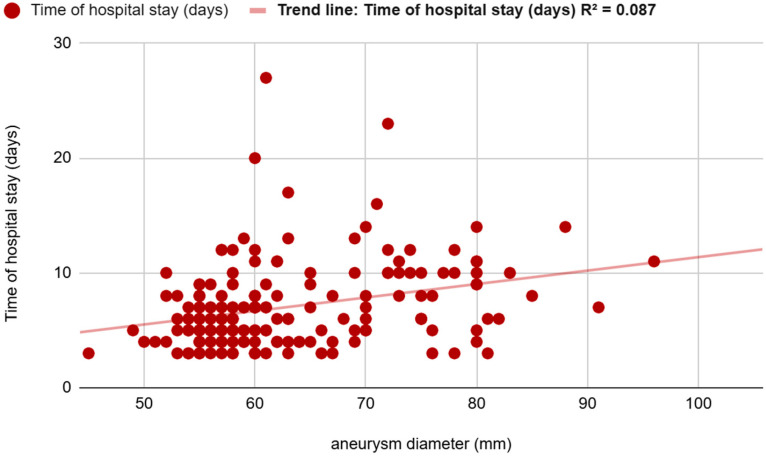
Correlation between aneurysm diameter and hospital stay in a linear regression model.

A linear regression model indicated a weak but positive correlation between aneurysm diameter and hospital stay (R^2^ = 0.087) (Figure 6).

## 4. Discussion

Even though EVAR has emerged as a less invasive alternative to open surgical repair for the treatment of AAA, the influence of preoperative aneurysm size on long-term outcomes remains unclear. The existing literature presents conflicting evidence; some studies suggest that larger aneurysms are associated with higher risks of complications, such as endoleaks, graft failure, and mortality, while others do not show a significant correlation between aneurysm size and adverse post-EVAR outcomes [6,7,8,15,16,17,18,19,20,21]; interestingly, some studies also suggest that an AAA with a larger bifurcation (≥20 mm) may be protective against limb graft occlusion, potentially due to improved flow dynamics and reduced kinking risk [22].

The proper selection of patients suitable for EVAR is crucial for reducing the risk of complications, reintervention rates, and mortality following the procedure [23,24]. Patients selected for EVAR are older, have a greater burden, and more comorbidities [25]. AAA size is also a factor considered in patient selection. When the size of an aneurysm increases, the sac expands both horizontally and vertically, which may lead to shortening of the aneurysm neck and cause difficulties during graft selection and implantation [26,27,28]. Although maximum diameter is a key selection criterion, anatomical complexity—such as neck tortuosity and iliac involvement—may co-occur with larger aneurysms and contribute to technical difficulty and adverse outcomes. Current guidelines recommend intervention for AAA at diameters ≥5.5 cm in men and ≥5.0 cm in women [13]; however, they are primarily based on rupture risk, with comparatively less emphasis placed on long-term morbidity and mortality following EVAR, and do not define an upper size limit of an aneurysm. To enhance the outcomes of patients undergoing EVAR and reduce the risk of the procedure, all possible risk factors need to be analyzed. The one that is elementary and can be easily measured is the preoperative aneurysm diameter.

In this single-centre study, we analyzed 176 patients who underwent EVAR. The smallest preoperative diameter was 45 mm, and the largest AAA was 105 mm. A patient with the smallest AAA was found to have coexisting aneurysms of both common femoral arteries: a 53 mm left common femoral artery aneurysm (LCFAA) and a 35 mm right common femoral artery aneurysm (RCFAA), with no significant comorbidities. Although the aneurysm was below the standard measurements threshold for intervention for AAA, it met the criteria of intervention for CFAA. As a result, the decision to proceed with endovascular repair was influenced by the concomitant presence of large bilateral common femoral artery aneurysms and low procedural risk. Statistical analysis revealed key predictors of complications. Patients in the top one-third of aneurysm size had significantly higher rates of all long-term adverse outcomes compared to those in the lower two-thirds, for late complications (*p* = 0.049) at 36 months. The largest aneurysms had a significantly higher rate of late endoleak (*p* = 0.041, OR = 5.365, 95% CI: 1.071–26.871). The correlation is even stronger for overall mortality (*p* = 0.022). In our group, patients with aneurysms the size of above 6.4 cm had higher rates of mortality compared to those with smaller aneurysm diameters. For early complications, increased risk was associated with branched grafts, including iliac branch devices (*p* = 0.008, OR = 5.884, 95% CI: 1.598–21.662). Late complications were significantly correlated with symptomatic presentation (*p* = 0.003, OR = 3.616, 95% CI: 1.533–8.529) and acute admission (*p* = 0.033, OR = 0.345, 95% CI: 0.130–0.916). The correlation between aneurysm diameter and hospital stay is weak(R^2^ = 0.087). A larger AAA diameter was not associated with a significantly longer length of hospital stay. Although a weak statistical correlation between aneurysm size and hospital stay was observed, this association is unlikely to be clinically relevant. Length of hospitalization following EVAR is predominantly driven by perioperative complications, frailty, and logistical factors, rather than aneurysm diameter itself.

In contrast to our results, some studies indicate that there is no significant correlation between preoperative aneurysm diameter and patient outcome following EVAR. For example, the work of Benjamin Ferrel shows that a larger primary aneurysm diameter was not related to higher mortality and more complications [6]. Diameter alone was not associated with poorer outcomes, and patients can undergo EVAR without concern for increased risk due to larger aneurysm size. However, Ferrel’s paper revolved only around patients with infrarenal AAA, with a mean follow-up period of 5.6 ± 3.5 years.

Jesse A. Columbo’s work revolves around patients unfit for surgery and in poorer health and suggests that to reduce AAA-related mortality in such patients, EVAR should be performed for AAAs with a size >6 cm in women and >8 cm in men. In this case, before AAA reaches that size, a watch-and-wait strategy should be considered due to EVAR itself being a preventive procedure [15].

In Hye, Robert J.’s research, there was a non-significant correlation between AAA size above 5.5 cm and increased risk of reintervention [16]. He compared aneurysm size with age; both of those factors are risk indicators alone, but not in combination.

Research by Pinar Ulug covering the subject of smaller AAAs (4.0 cm to 5.5 cm) shows that there is no evidence of an advantage to early repair for both open and endovascular methods [17].

Other papers, similarly to what we found, suggest that AAA size is one of the key predictors of patients’ outcomes and survival.

Sooyeon Kim’s paper found that patients with AAA sizes smaller than 5.5 cm had better 5-year survival compared to those with larger aneurysm diameters [18].

The work of Mária Rašiová suggests that the mortality of patients following EVAR is higher in patients with increasing diameter and volume, both before and after the procedure [19]. Higher mortality was also correlated with failure of sac regression and anticoagulation therapy.

Multiple studies have investigated the relationship between aneurysm size and outcomes following EVAR, with consistent evidence indicating that aneurysm diameter plays a significant role in prognosis. For instance, research by Haekyung Jeon-Slaughter demonstrates that mid-term survival after EVAR is significantly and independently associated with AAA size, even after adjusting for comorbidities [7]. Interestingly, preoperative AAA size in that study was not an independent predictor of long-term mortality. The aneurysm size did not remain a statistically significant element impacting survival over time, suggesting that additional factors—both clinical and anatomical—are likely to contribute to late outcomes. This complexity indicates that while aneurysm size is important, it cannot be the sole parameter guiding post-EVAR risk assessment.

Montelione’s work extended this perspective by showing that worse long-term outcomes were associated not only with larger aneurysm diameter but also with total aneurysm volume and the absence of favorable early sac remodeling [8]. These three factors—diameter, volume, and remodeling—were all independently linked to increased mortality and reintervention rates. This implies that measuring aneurysm volume and assessing early post-procedural remodeling may provide a more accurate risk stratification than relying on diameter alone. Incorporating these variables into surveillance protocols may help detect high-risk patients earlier and consequently adjust follow-up intensity.

Anatomical complexity has also been strongly linked to patient outcomes in Welborn’s paper. The study proposed that smaller aneurysms are typically associated with more favorable anatomy—such as longer proximal necks and less vessel tortuosity—resulting in improved outcomes after EVAR [20]. Larger aneurysms, by contrast, are often characterized by challenging anatomical features that negatively affect stent-graft fixation and seal integrity.

These findings are reflected by Zarins and co-authors, who reported a stepwise increase in AAA-related mortality over a five-year period: patients with aneurysms <5.0 cm in size had the lowest risk, those with aneurysms 5–6 cm in size had intermediate risk, and those with aneurysms ≥6.0 cm in size experienced the highest mortality [21]. Within the latter group, 8% of patients died from aneurysm-related causes. These data reinforce the importance of aneurysm diameter as a determinant of long-term outcome.

Further evidence from Huang [29] confirms that patients with large AAAs (≥6.0 cm) face significantly higher rates of all-cause mortality, complications, and reinterventions following EVAR. However, as Jones points out, some of the survival advantage observed in patients with smaller aneurysms may also be attributed to demographic and clinical differences [30]. Their analysis showed that these patients were generally younger and had fewer comorbidities, further complicating the interpretation of size as an isolated risk factor. This underlines the importance of integrating clinical context into decision-making when evaluating EVAR candidates.

Finally, the study by Oliveira identified an aneurysm diameter >70 mm as an independent anatomical predictor of all-cause mortality. Interestingly, only a small proportion of deaths were aneurysm-related; however, most were attributed to cardiovascular causes [31]. This finding suggests that while aneurysm size correlates with procedural complexity and anatomical risk, many deaths during extended follow-up may be more closely linked to the patient’s overall cardiovascular health than to EVAR failure itself. It emphasizes the need for a holistic patient assessment, combining anatomical risk factors with systemic health indicators to optimize outcomes.

Higher mortality in patients with larger aneurysms can be explained by the fact that larger aneurysms have more complex anatomy in general. This happens because the AAA sac expands not only by increasing in diameter but also changing size longitudinally, leading to shortening of the aneurysm neck, causing greater tortuosity and expansion into common iliac arteries which directly causes worse stent-graft fixation.

Aneurysm size could be incorporated as a predictor of long-term prognosis following EVAR and become an indication for more frequent follow-up for high-risk patients. Patients with large aneurysms may benefit from closer post-procedural surveillance and consideration for more robust fixation strategies, including fenestrated or branched devices in borderline anatomies. Although more frequent imaging and follow-up visits increase short-term healthcare costs, early detection of complications such as endoleaks or graft migration can prevent catastrophic events, such as aneurysm rupture or the need for emergency reinterventions, which are far more resource-intensive.

To summarize, most authors suggest that primary AAA diameter is an important predicting factor of mortality and morbidity in patients following EVAR; the differences mainly concern cut off of the size where the risk is the highest and if the size alone is a key risk factor. We tend to conclude that preoperative aneurysm diameter is essential for predicting, and most important for reducing the risk of complications, especially endoleaks and the need for reoperation in patients undergoing EVAR. Proper selection of candidates for a procedure is necessary to achieve the best possible results without burden for a patient, covering complications, health impairment, and eventually death.

This work builds upon prior research suggesting that aneurysm size should be considered not only as a rupture risk marker, but also as an important predictor of late complications and mortality following EVAR. Unlike current guidelines, which do not specify maximal aneurysm diameter, our findings indicate that patients with large aneurysms may benefit from earlier, more intensive post-procedural surveillance and preoperative consideration for fenestrated and branched devices regarding complex aneurysm anatomy. By classifying patients based on preoperative aneurysm diameter into tertiles, we demonstrate that aneurysm size correlates with adverse long-term outcomes, particularly late endoleak and overall survival, pointing out the need for more individualized, anatomy-based treatment, focusing on patients with larger aneurysms.

Despite the valuable insights from our work, this study has several limitations that may affect the interpretation and generalizability of the findings. Firstly, the research was retrospective and conducted at a single medical center, involving a relatively small patient cohort. Secondly, the study lacked data on aneurysm sac regression post-EVAR and long-term device integrity, limiting the assessment of long-term outcomes. Additionally, anatomical variability, including differences in aortic neck morphology and iliac anatomy, was not quantified; the measurements focused solely on aneurysm diameter. Future research should adopt prospective, multicenter study designs with larger patient populations to improve the generalizability of findings. Long-term follow-up, including assessment of aneurysm sac regression and device performance, would provide a more complete understanding of EVAR durability. In addition, detailed evaluation of anatomical characteristics such as aortic neck and iliac morphology could enhance patient selection and risk stratification, ultimately helping to address current limitations and improve clinical outcomes.

## 5. Conclusions

Preoperative aneurysm diameter is a strong predictor of late complications and mortality following EVAR, particularly endoleaks and reinterventions. Patients with larger aneurysms may benefit from more intensive post-procedural surveillance and, in selected cases, advanced endovascular strategies such as fenestrated or branched devices. These findings support a more individualized, anatomy-driven approach to EVAR that goes beyond current guideline recommendations and emphasizes careful patient selection to optimize long-term outcomes.

## Figures and Tables

**Table 1:** Summarized comorbidities of patients and their percentage distribution.

Comorbidity	Number of Cases	Percentage of Patients with Each Comorbidity
CAD (Coronary Artery Disease)	66	37.5%
History of MI (Myocardial Infarction)	59	33.5%
Coronary Revascularization	62	35.2%
Hypertension	140	79.5%
DM (Diabetes Mellitus)	48	27.3%
Hyperlipidemia	57	32.4%
HF (Heart Failure)	22	12.5%
COPD (Chronic Obstructive Pulmonary Disease)	6	3.4%
PAD (Peripheral Artery Disease)	17	9.7%
CKD (GFR < 30)	10	5.7%
Dialysis	0	0%
Neurological (TIA, Stroke)	30	17%
Thyroid Disease	15	8.5%
Liver Failure	0	0%
Smoking	107	60.8%

**Table 2:** Number and percentage distribution of different types of procedures performed.

Procedure Type	Number of Procedures	Percentage (%)
EVAR	97	55.11
pEVAR	64	36.36
EVAR + Iliac Branch	12	6.82
pEVAR + Iliac Branch	3	1.70
**Total**	**176**	**100**

## Data Availability

The data presented in this study are available on request from the corresponding author. The data are not publicly available due to privacy.

## Abbreviations

The following abbreviations are used in this manuscript:

AAA	Abdominal Aortic Aneurysm
EVAR	Endovascular Aneurysm Repair
RCFAA	Right Common Femoral Artery Aneurysm
LCFAA	Left Common Femoral Artery Aneurysm
CFAA	Common Femoral Artery Aneurysm
CT	Computed Tomography
CTA	Computed Tomography Angiography
CAD	Coronary Artery Disease
DM	Diabetes Mellitus
HF	Heart Failure
COPD	Chronic Obstructive Pulmonary Disease
PAD	Peripheral Artery Disease
CKD	Chronic Kidney Disease
TIA	Transient Ischemic Attack
pEVAR	Percutaneous Endovascular Aneurysm Repair
PTA	Percutaneous Transluminal Angioplasty
SD	Standard Deviation
IQR	Interquartile Range
MI	Myocardial Infarction
